# Characterization of Alzheimer’s Disease‐Related Plasma Biomarkers in Gammaherpesvirus Infected Common Marmosets

**DOI:** 10.1002/alz.092787

**Published:** 2025-01-03

**Authors:** Stacey L Piotrowski, Amanda Lee, Allison Tucker, Heather Narver, Brianne Stein, Julia Oluoch, Ting Zhang, Lauren K Hayrynen Schaeffer, Afonso C Silva, Gregory W Carter, Steven Jacobson, Stacey J Sukoff Rizzo

**Affiliations:** ^1^ National Institute Neurological Disorders and Stroke (NIH NINDS), Bethesda, MD USA; ^2^ Purdue University, West Lafayette, IN USA; ^3^ University of Pittsburgh School of Medicine, Pittsburgh, PA USA; ^4^ The Jackson Laboratory, Bar Harbor, ME USA

## Abstract

**Background:**

The infectious hypothesis of Alzheimer’s disease (AD) suggests that microbes may play a role in pathogenesis by triggering the pathologic cascade or contributing to disease progression. Herpesviruses, such as Epstein‐Barr virus (EBV), have been of high interest in AD and related neurodegenerative diseases, in part due to their ability to establish lifelong latent infection and potentially reactivate. However, further research is needed to fully understand the role of herpesviruses in these diseases. The common marmoset *(Callithrix jacchus)* is not only a translationally relevant model of AD, but also herpesvirus infection. Callitrichine herpesvirus 3 (CalHV‐3) is a naturally occurring gammaherpesvirus in the marmoset that is phylogenetically and biologically similar to EBV. This study aimed to understand the contribution of persistent gammaherpesvirus infection to AD‐related pathology in the common marmoset.

**Methods:**

Whole blood from over 200 animals was collected from the NIH NINDS colony and the MARMO‐AD colony at the University of Pittsburgh during routine health surveillance and experimental procedures. Plasma was evaluated using MesoScale Discovery (MSD) multi‐plex ELISA Aβ peptide panel 4G8 and Neurology Panel 1 (GFAP, Neurofilament L, Total Tau). Peripheral blood mononuclear cells (PBMCs) were isolated using lymphocyte separation media. DNA was extracted from PBMCs and screened for CalHV‐3 using a droplet digital PCR (ddPCR) assay that amplified the virus and a housekeeping gene to allow for the quantification of viral loads (copies of CalHV‐3/10^6^ cells).

**Results:**

20‐30% of the tested colonies were positive for CalHV‐3, with a range of viral loads and the highest prevalence in aged animals (≥8 years old). There was significantly higher plasma Neurofilament L (NfL) and total tau in virally infected aged animals compared to uninfected aged animals.

**Conclusions:**

The enhancement of plasma biomarkers, such as NfL and tau, in gammaherpesvirus infected aged marmosets suggests that viral infection may contribute to AD‐related pathology and neurodegeneration. Investigation is ongoing in additional animals to further characterize the relationship of plasma biomarkers to viral infection and viral load. The common marmoset is a unique translational model that allows for the investigation of AD and the potential role that herpesviruses play in disease manifestation and progression.

